# Thank You to Our Reviewers

**DOI:** 10.1017/cts.2020.502

**Published:** 2020-07-07

We are indebted to the expert referees who have volunteered their time to review submissions for the *Journal of Clinical and Translational Sciences* in 2019. The editors, authors and readers are immensely grateful for their thoughtfulness and expertise that have served our journal well.

Stephanie Abbuhl
Terry Ainsworth
Myles Akabas
Tabia Akintobi
Bianca Albers
Emily Anderson
Joseph E. Andrews
Judith Aronson
Jane Atkinson
Mona AuYoung
Lorena Baccaglini
Laura Balis
Makenzie L. Barr
Mark Bauer
Ana Baumann
Liza Behrens
Miriam Bender
L. Michelle Bennett
Jesse David Berman
Nazleen Bharmal
Arlene Bierman
Beatrice A. Boateng
Kathleen T. Brady
Patrick Brandt
Donna Brassil
Tabetha A. Brockman
Chad Brummett
Cheryl Bushnell
Nancy A. Calvin-Naylor
Jose Cancelas
Marjory Charlot
Michael Charlton
Peggy Chen
James J. Cimino
Robert A. Clark
Barry Coller
Linda Cottler
Mara Coyle
Jennifer Croker
Orianna Damas
Colin A. Depp
Megan Doerr
Aalap Doshi
Ann M. Dozier
Milton Mickey Eder
Vicki L. Ellingrod
Kolaleh Eskandanian
Estela Estape
Alecia Fair
Stephanie Freel
Janice L. Gabrilove
Russell E. Glasgow
Melody Goodman
Mary Gorfine
Shelly Gray
Alexandra Greenberg-Worisek
Matthew Grossman
Kristie B. Hadden
Heidi Hanson
Brett Harnett
Paul A. Harris
Matthew Hart
J.R. Haywood
William R. Hogan
Mike Hogarth
Lianna Ishihara
Rebecca D. Jackson
Jason Johnson
Rachel Jones
Yvonne Joosten
Felichism W. Kabo
Allison Karpyn
Heidi Keeler
Matthew Keener
Philip Kern
Roohi Kharofa
Dana King
Agnes Kiragga
Jackie Knapke
Ron Koenig
H. Robert Kolb
Rhonda G. Kost
Sunil Kripalani
Jack Kues
Danielle Lavallee
Colleen E. Lawrence
Scott P. Layne
Scott Leischow
Aaron L. Leppin
Vivian Lewis
Z.N. Li
Mike Linke
Kanchan Lota
Mike Lyons
Jane Mahoney
Elizabeth Malcolm
Camille Anne Martina
George A. Mashour
Phoenix A. Matthews
Colleen A. Mayowski
Wayne T. McCormack
Rachel McGarrigle
Emma Anne Meagher
Tara G. Mehta
Paul Meissner
Jonathan Merrell
Frederick J. Meyers
Peter Meyers
Lloyd Michener
Christopher Miller
Rachel Moon
Sean Mooney
Christopher Morley
Cynthia Morris
Gia Mudd–Martin
Donald E. Nease
John Oetzel
Christian Ohmann
Janet M. Okamoto
Julie Ozier
Dinesh Pal
Angela L. Palmer–Wackerly
Cecilia Patino Sutton
Susan M Perkins
Sharon Plon
Byron Powell
Lori Lyn Price
Enola Proctor
Jill M. Pulley
Boriska Rabin
Julie Rainwater
Marie Rape
Jennifer Reed
Robert L. Rhyne
Suzanne Rivera
Brenda Robles
Stephen Joseph Rosenfeld
Kristie Ross
Doris Rubio
Patrick Ryan
Maritza Salazar Campo
Elias Samuels
Elaine Schattner
Michael Schembri
Cari Schmidt
Ellie Schoenbaum
Linda M. Scholl
Harry Selker
Dana Shaffer
Jackilen Shannon
Anantha Shekhar
Greg Simon
Christine Sorkness
Kathleen R. Stevens
William Stratbucker
Alisa Surkis
Alan Tait
Ellen Tambor
Velma Thompson
Jonathan Tobin
Joel Tsevat
Katherine R. Tuttle
Jason Umans
Laurie Van Egeren
Melissa Van Dyke
Boris Volkov
Rahma Warsame
Kevin J. Weatherwax
Karen Weavers
Fern Webb
Mary Beth Weber
Terrence Witt
Henry Nolan Young

